# Spatiotemporal cell landscape of human embryonic tooth development

**DOI:** 10.1111/cpr.13653

**Published:** 2024-06-12

**Authors:** Yueqi Shi, Yejia Yu, Jutang Li, Shoufu Sun, Li Han, Shaoyi Wang, Ke Guo, Jingang Yang, Jin Qiu, Wenjia Wei

**Affiliations:** ^1^ Department of Stomatology, Tongren Hospital Shanghai Jiao Tong University School of Medicine Shanghai China; ^2^ State Key Laboratory of Oral Disease, West China Hospital of Stomatology Sichuan University Chengdu China; ^3^ Hongqiao International Institute of Medicine Tongren Hospital, Shanghai Jiao Tong University School of Medicine Shanghai China; ^4^ Department of Obstetrics and Gynecology, Tongren Hospital Shanghai Jiao Tong University School of Medicine Shanghai China; ^5^ Department of Oral Surgery, Shanghai Ninth People's Hospital Shanghai Jiao Tong University School of Medicine Shanghai China

## Abstract

Understanding the cellular composition and trajectory of human tooth development is valuable for dentistry and stem cell engineering research. Previous single‐cell studies have focused on mature human teeth and developing mouse teeth, but the cell landscape of human embryonic dental development is still unknown. In this study, tooth germ tissues were collected from aborted foetus (17–24 weeks) for single‐cell RNA sequence and spatial transcriptome analysis. The cells were classified into seven subclusters of epithelium, and seven clusters of mesenchyme, as well as other cell types such as Schwann cell precursor and pericyte. For epithelium, the stratum intermedium branch and the ameloblast branch diverged from the same set of outer enamel‐inner enamel‐*ALCAM*+ epithelial cell lineage, but their spatial distribution of two branches was not clearly distinct. This trajectory received spatially adjacent regulation signals from mesenchyme and pericyte, including JAG1 and APP. The differentiation of pulp cell and pre‐odontoblast showed four waves of temporally distinct gene expression, which involved regulation networks of LHX9, DLX5 and SP7, and these genes were regulated by upstream ligands such as the BMP family. This provides a reference landscape for the research on early human tooth development, covering different spatial structures and developmental periods.

## Graphical abstract

This study integrated single‐cell transcriptome and spatial transcriptome to depict the developmental trajectory of epithelial and mesenchymal cells in human foetal tooth.

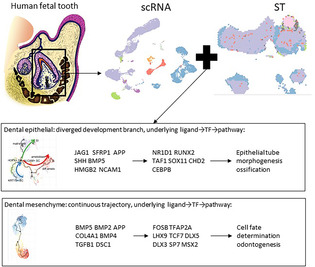

## 1 INTRODUCTION

Human tooth development is a long and complex process that begins during the embryonic stage and continues until adolescence. This process represents a good model of organogenesis involving epithelial–mesenchyme interaction, morphogenesis and adult tissue self‐renewal.^1^ During the embryonic stage, dental development begins with the formation of mesenchyme from the neural crest,^2^ and the dental placode originates from the thickened oral epithelium. During this stage, the dental epithelium starts to proliferate in some regions of the dental lamina, then migrates to the deep connective tissue, and the end‐stage epithelium proliferates and further differentiates to form the enamel organ. Simultaneously, the ectomesenchyme layers beneath the proliferative epithelium also rapidly proliferate and aggregate around the epithelial layer to form the condensed mesenchyme layer. The epithelia and mesenchyme that proliferate in this restricted region together consist in the tooth germ,^3^ which is the structural basis of further dental development. The tooth germ is made up of three parts: (1) The oral ectoderm‐originated enamel organ, which gives rise to enamel; (2) The ectomesenchyme‐originated dental papilla tissue, which gives rise to dentin and pulp tissue; (3) The ectomesenchyme‐originated dental follicle, that gives rise to cementum, periodontal ligament and alveolar bone.^4^ Attaining a comprehensive comprehension of dental development necessitates a meticulous cell atlas encompassing the entirety of these heterogeneous cell types and tissues.

The rapid development of single‐cell techniques provides a new opportunity for this task.^5^ Recent studies on mouse dental tissues using single‐cell analysis have elucidated the heterogeneity of cells from early‐state cranial neural crest cells^6^ to later incisor tissues,^7^, ^8^ and delineated the differentiation and migration trajectory of epithelial and mesenchymal cells. Other studies have also constructed a landscape of postnatal human dental pulp,^9^, ^10^ apical papilla,^7^ periodontal tissues^9^, ^11^ and immature tooth germ,^12^ using dental tissues from healthy volunteers. Based on these and other existing data, Hermans et al.^13^ constructed a comprehensive dental cell landscape that based on existing data. The landscape covers various cell types of mouse incisor and molar tissue as well as human molar tissue during adolescence and young. This work has proven which has been proved valuable for clinical and bioengineering studies. However, some challenges remain unsolved. Firstly, these resources could not replace human prenatal dental tissues data, as mouse incisors have an everlasting self‐renewal capacity and exhibit significant physiological differences compared with humans.^14^ A recent study by Alghadeer et al. used scRNA sequencing on human embryonic enamel tissues, and established human‐specific in vitro enamel models for clinical studies.^15^ This study demonstrated the unique value of single‐cell data on human‐developing dental tissues. However, a drawback is the insufficient spatial resolution of current single‐cell analysis relied on immunofluorescence that tags one or two proteins to study the spatial distribution of cell subtypes. This procedure inevitably misses more refined information on the developmental process based on multiple genes.

To elucidate these aspects, we obtained samples of primary and permanent tooth germ tissues from aborted foetuses aged between 17 and 24 weeks post‐conception (PCW). Subsequently, we then used single‐cell RNA sequencing (scRNA) and spatial transcriptome (ST) technologies to create a comprehensive spatiotemporal cell landscape. Our study aimed to address the following questions. First, what is the cellular composition of human embryonic tooth germ at different stages? Second, what is the developmental trajectory of dental epithelium and mesenchyme, as well as their underlying biological process, gene regulatory networks, and intercellular signalling pathways? Third, how does these elements spatially distribute? This cell atlas could serve as a reference for future functional and analytical studies in oral medicine and developmental biology.

## 2 METHOD

### 2.1 Sample collection and preprocessing

This study is approved and supervised by the ethic committee of Shanghai Tongren Hospital. Five fresh human foetal tooth germ tissues were collected at different embryonic developmental stages (2, 2 and 1 samples at 17, 20 and 24 PCW, respectively). Informed consent was obtained from each donor before the termination of pregnancy. All prenatal examinations showed that the foetus had no developmental abnormalities. The method of pregnancy termination was medically induced. The parents of the foetus are healthy, with no family history of facial and dental developmental abnormalities. Each sample was processed in sequenced separately. Details of sample pre‐process was provided in Data S1.

### 2.2 scRNA and ST

Library construction, sequencing and data filtration were described in the Supplementary methods in Data S1. Both libraries of scRNA and ST were sequenced with paired‐end 150 bp sequencing (PE150) by NovaSeq 6000 platform. We retained non‐doublet cells with number of detected genes >500 and <2500, and mitochondria gene percentage <5%. Library size normalization was performed with NormalizeData function in Seurat^16^ to obtain the normalized count. Specifically, the global‐scaling normalization method ‘LogNormalize’ normalized the gene expression measurements for each cell by the total expression, multiplied by a scaling factor (10,000 by default), and transformed by Seurat: SCTransform function. Cell phase was defined by Seurat: CellCycleScoring function based on cell cycle gene list.^17^ Spatial transcriptome data were pre‐processed by Seurat in a similar way. We observed a median of 1378 genes and 250,835 reads per spot (1892 spots in total), where 95.9% barcodes and 97.0% UMIs were valid.

### 2.3 Cluster analysis

For scRNA data, we applied Louvain community detection‐based cluster analysis implemented in Seurat: FindClusters function with number of principle component = 20 and resolution = 0.5. We applied Seurat: FindMarkers Wilcoxon test to find top 15 markers for each cluster, and defined them according to existing knowledge on dental cell types. Epithelium and mesenchyme cell were further extracted and underwent a second round of clustering, with the same parameters.

### 2.4 Pseudotime analysis

For epithelium and mesenchyme (excluding isolated follicle and MSC clusters) cell groups, we applied monocle3:learn_graph and order_cells function^18^ to learn the pseudotime trajectory. We then applied monocle3: graph_test function on top 5000 variable genes detected by Seurat: FindVariableGenes function to find all genes that significantly altered along the trajectory. Genes with FDR‐adjusted *p* < 0.01 were further used for module detection by monocle3: find_gene_modules function. For the ease of interpretation, in epithelium cell group we separately applied module detection in each branch, and only retained gene modules that showed up‐regulation or down‐regulation along each of the three branches.

### 2.5 Transcription factor regulation network analysis

We applied SCENIC^19^ to construct transcription factors (TF)‐target genes regulation network, separately for epithelium and mesenchyme. TF‐motif annotation was downloaded from SCENIC website, which was corresponded to hg38 refseq gene annotation and contained a 10 kb window of each gene. We preserved the target genes pertaining to each transcription factor's extended network and subsequently subjected the module genes identified through pseudotime analysis to a Fisher test, thereby determining whether these genes exhibited enrichment within any of the transcription factor regulation networks. TFs with FDR‐adjusted *p* < 0.01 and odds ratio >2 were retained for each module. We further extracted the overall expression of a TF and all its targets (so‐called regulon) of each cell to verify that this expression value had a similar expression pattern as module genes. For epithelial SCENIC analysis result, we removed TF which was expressed in <25% of cells from the corresponding branch. For mesenchyme SCENIC result, we removed TF expressed in <25% of cells in the pulp‐odontoblast trajectory.

### 2.6 Gene Ontology analysis

We used ClusterProfiler R package^20^ to apply Gene Ontology‐Biological Process (GO‐BP) analysis by a hypergeometry test. The background gene list was set as all genes with GO‐BP annotations. Only terms with >5 and <500 genes were considered. We applied simplify function to remove highly similar terms. Only terms with FDR‐adjusted *p* < 0.01 were retained. The odds ratio and *p*‐value were reported in the word cloud plot.

### 2.7 The nichenetr analysis

We used nichenetr R package^21^ to apply signalling network analysis as described previously.^12^ We separately considered (1) up‐regulation modules of each of the three epithelium branches; (2) immature (first and second wave) and mature (fourth wave) of mesenchyme differentiation as target gene list, and the final signalling network were combined in each of these two categories. Ligands were prioritized by area under curve (AUC) and Pearson correlation coefficient calculated by nichenetr.

### 2.8 Spatial transcriptome analysis

After defining cell clusters on scRNA data, we projected it to spatial transcriptome data by Seurat: FindTransferAnchors and TransferData functions to estimate the cell type probability of each spot. We assign cell types with the highest probability to each spot. We used misty R package^22^ to estimate the spatial relationship among highlighted cell types, TFs and expression levels of ligands and target genes. We chose the ‘intra’ mode in misty analysis and trained a linear model to predict each of the target values (cell probability or gene expression) by adjacent predicting values. Spatial adjacency was quantified by importance score in the prediction model.

## 3 RESULTS

### 3.1 Overview of cell types in human embryonic tooth

**FIGURE 1:**
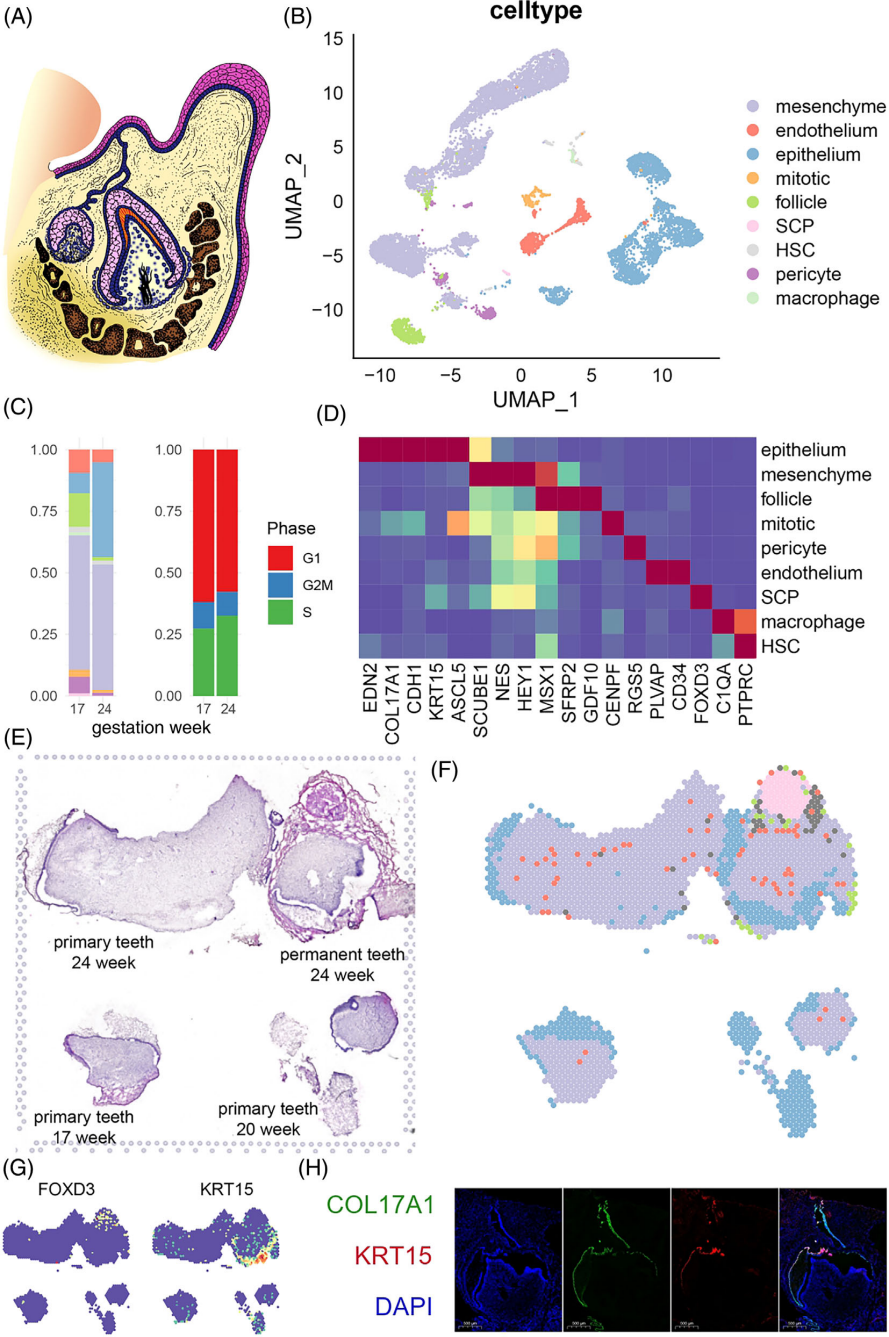
Overview of the human embryonic tooth cell atlas. (A) schematic diagram of structure of primary and permanent tooth germ of 20 weeks. (B) UMAP plot of single‐cell clustering. AP, apical papilla; NCC, neural crest cell. (C) percentage of each cell type and cell cycle phase in samples from different gestational weeks. Cell cluster colour is corresponded to B. (D) Marker scaled expression of each cluster. Mean expression level per cluster was scaled to 0–1 and shown as colour of each grid. (E) H‐E staining of spatial transcriptome slice. (F) Cell type annotation of each spot, defined as the cell type with the highest probability. Cell cluster colour is corresponded to B. (G) Marker scaled expression of cell markers. (H) Immunofluorescence of COL17A1 and KRT15.

We collected tooth germ tissues from aborted foetuses spanning 17–24 PCW (Figure 1A) for scRNA and ST. In scRNA, we obtained expression data for 11,218 single cells of nine general cell types that passed quality control (Figures 1B–D and S1). The cell groups were classified based on gene panel (Table S1) from existing cell atlas of mouse tooth,^7^, ^8^ adolescent tooth^9^ and histology knowledge (Figures 1D and S1). Cells with epithelial origin were defined based on the expression of dental epithelial markers *KRT5*, *KRT14*, *FXYD3* and *COL17A1* (Figures 1D and S1). Mesenchyme origin cells were characterized by their expression of *EMILIN1* and *MSX1*. A subgroup of mesenchyme origin cells expressing SFRP2 was defined as apical pulp‐like cells. Lastly, we defined mitotic cells, pericytes, endothelium, neural crest cells (NCC), macrophages and lymphocytes based on their specific markers: *CENPF*, *RGS5*, *CD34*, *FOXD3* + *SOX10*, *C1QA* and *PTPRC* + *CD52*, respectively (Table S1). Across different gestational weeks, we observed differences in the proportion of epithelium and pericytes, but no differences in cell cycle distribution (Figure 1C). We conducted a differential expression analysis between developmental time points for pericytes, and found minimal differences with biological significance. This result indicated that pericytes did not show significant differentiation between the two time points, thus we mainly focused on dental epithelium and mesenchyme clusters.

ST was applied to tooth slices from three donors: a slice from 24 PCW embryo containing both the tooth germ of the primary tooth and the tooth germ of the permanent tooth, a slice from a 17 PCW primary tooth and a slice from a 20 PCW primary tooth (Figure 1E). The proportion of each cell cluster of scRNA in each spatial plot was evaluated using the anchor‐based integration method implemented in Seurat v3 (Figures 1F and S2). As expected, we observed that the epithelium incompletely surrounded the pulp tissues, which consisted mainly of mesenchyme cells, scattered pericytes and endothelium in the 24‐week sample. AP‐like cells were only observed in the permanent tooth germ of 24‐week sample. We also found an isolated NCC group surrounded by endothelium (Figure 1F). This group was adjacent to the permanent tooth germ and likely represented the process of neuron outgrowth. The spatial distribution of cell markers at the transcription and protein levels, such as *KRT15*, *COL17A1*also supported these spatial characteristics (Figure 1G,H). Taken together, we successfully constructed a cell type atlas of the human embryonic tooth germ that covers various developmental timepoints and spatial structures.

### 3.2 Branched trajectory of dental epithelium differentiation

**FIGURE 2:**
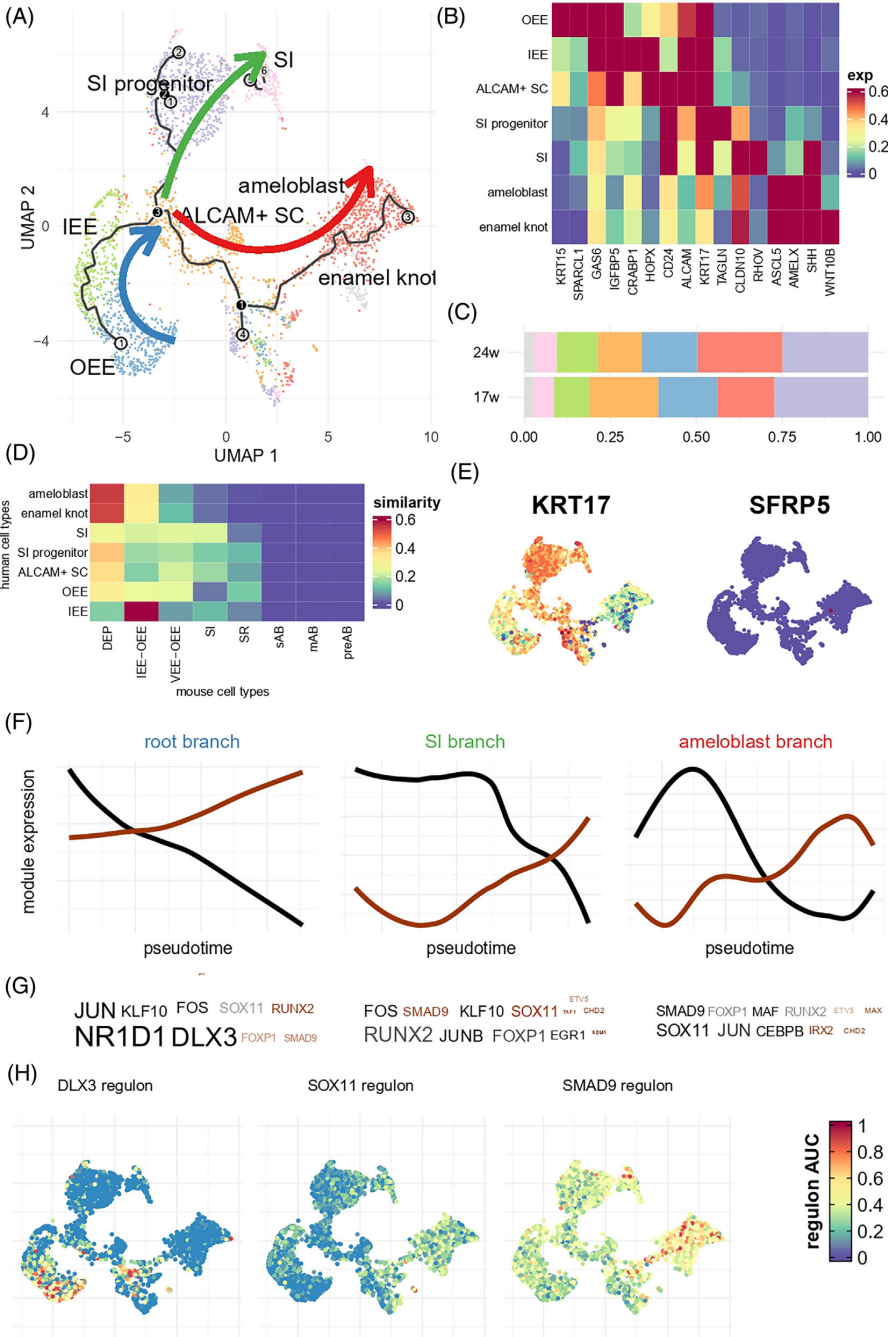
In‐depth analysis of dental epithelium. (A) UMAP plot and pseudotime analysis of epithelium cell clusters. Arrows marked development direction. EK, enamel knot; IEE, inner enamel epithelium; OEE, outer enamel epithelium; SC, epithelial stem cell; SI: stratum intermedium. (B,C) Similar to Figure 1C,D, but for epithelium clusters. (D) Similarity between epithelial clusters from human and mouse embryonic tooth. DEP, dental epithelial progenitor; mAB, mature ameloblast; preAB, pre‐ameloblast; sAB, secretary ameloblast; SR, stellate reticulum. (E) expression level of human‐specific SI marker KRT17 and mouse‐specific DEP marker SFRP5. (F) Module expression along pseudotime. For each branch, we identified an up‐regulation module and a down‐regulation module. (G) Transcription factors whose targets were enriched for module genes. Brown colour denoted regulon enriched in ascending module, and black colour denoted regulon enriched in descending module. Transparency showed enrichment *p*‐value (lower *p*‐value corresponded to lower transparency). Larger font size corresponded to higher odds ratio. Only TF that showed non‐zero expression were showed. (H) Regulon expression of selected TF.

Next, we conducted an in‐depth analysis of the cell constitution and developmental process of epithelial cell groups. By applying cluster analysis and pseudotime analysis, we partitioned all the epithelial origin cells into three branches (Figure 2A). The root branch expressed *GAS6* and *IGFBP5*, and consisted of two continuous cell clusters: outer enamel epithelium expressing *KRT15* and *SPARCL1* (OEE, Figure 2B), and inner enamel epithelium expressing *CRABP1* and *HOPX* (IEE). Most of the 17w epithelial cells were grouped into a root branch, supporting that root branch was the origin of epithelial development (Figure S3). The root branch ended at a cluster of *ALCAM*‐positive cells that also expressed *CD24* and *HOPX*, which were associated with epithelial stem cells. The *ALCAM*+ cell then gave rise to two branches. The first one was the SI branch consisting of SI progenitors expressing *KRT17* and *TAGLN* and stratum intermedium expressing *CLDN10*, which was further validated by immunofluorescence (Figure S3). The second was the ameloblast branch expressing *ASCL5* and *AMELX*. Lastly, we found a small subcluster of isolated cells near ameloblast expressing *SHH*, *WNT10A*, and exclusively *WNT10B*, which we defined as enamel knot (EK). Twenty‐four PCW tooth germ contained moderately more ameloblasts and fewer *ALCAM*+ SC than 17 PCW tooth germ, and the overall cell proportions were similar (Figure 2C). These results suggested that the development of SI and ameloblast have a divergent trajectory, even though they share the same set of OEE‐IEE lineage as progenitor. This result resembled previous identification of a dental progenitor that could give rise to both SI and ameloblast after injury,^23^ and suggested that the *ALCAM*+ population in our study might have similar roles as dental progenitors.

We further compared our human embryonic data with mouse data meta‐analysed by Hermans et al.^13^ using anchored‐based integration. Figure 2D shows profound cross‐species differences in expression profile, highlighting the necessity to study human tissues in dental developmental biology. Many human cell types mostly resembled dental epithelium progenitor (DEP), and were dissimilar to their corresponding cell types in mouse molars and incisors except for the IEE. We also found no similar cell types to any of the mouse ameloblast subtypes (pre‐ameloblast, secretary ameloblast and mature ameloblast). In fact, human ameloblast and enamel knot were mostly similar to DEP in mouse. There were also considerable differences in cell marker expression. In humans, *KRT17* (Figure 2E) was highly expressed in multiple epithelia, especially the SI lineage, whereas in mice, there were no notable expression patterns. On the other hand, although most human epithelia were similar to mouse DEP, the marker *SFRP5* (Figure 2E) exhibited minimal expression in human dental epithelium.

Finally, we analysed the biological processes underlying these branches. Using a threshold of FDR‐adjusted *p* < 0.01, we found 622, 1397 and 842 genes whose expression levels significantly altered along pseudotime in the root, SI and ameloblast branch, respectively. The gene module detection algorithm implemented in monocle3^18^ to find genes that were consistently up‐ or down‐regulated in each branch (Tables [Link], [Link]). SCENIC^19^ was then used to identify transcription factors (TF) that regulate these genes. TFs like *NR1D1*, *DLX3*, *RUNX2*, *SOX11* and *SMAD9* were predicted to regulate a large number of genes showing alterations in each branch (Figure 2F and Tables [Link], [Link]). For example, among 18 target genes of *DLX3*, *DLX*14 were down‐regulated in the root branch (Odds ratio, OR = 102, Fisher test *p* = 6.45 × 10^−18^). Consistently, the overall expression levels of *DLX3* and its targets (so‐called regulon^19^) were highly expressed in the primitive OEE, and gradually diminished in the root branch (Figure 2G). We highlighted regulons of several other TFs showing consistent expression patterns in SI and ameloblast branches, such as *SOX11* and *SMAD9* that were activated during SI and ameloblast development (Figure 2H).

### 3.3 Spatial patterns of dental epithelium development

**FIGURE 3:**
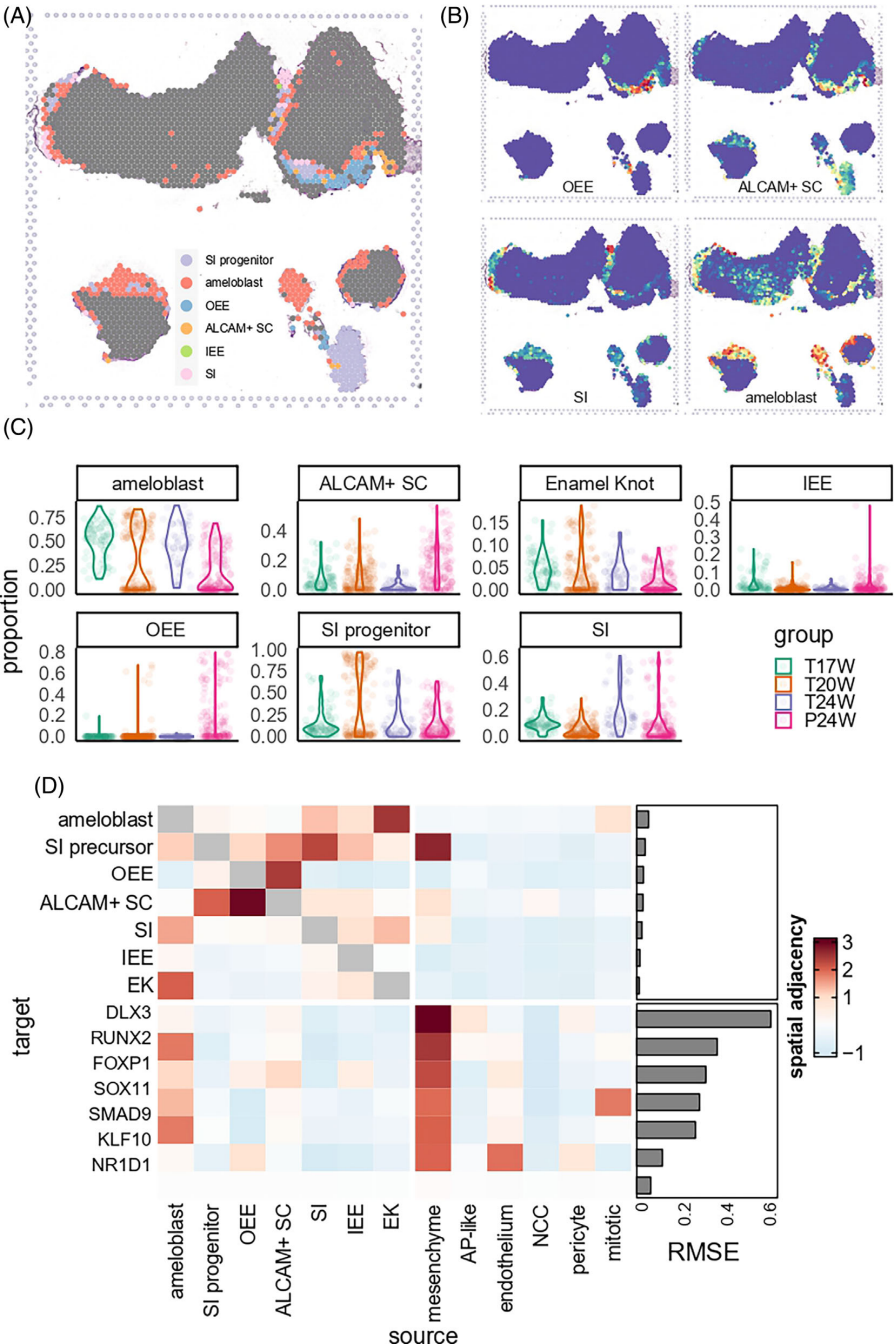
Spatial transcriptome of dental epithelium. (A) Cell type annotation of each spot. Spots not annotated as epithelial cell types were marked by dark grey. (B) Cell type probability spatial distribution. (C) Cell type probability distribution per developmental stage. (D) misty result of spatial adjacency, quantified by model importance score. Each grid showed the spatial adjacency between the source (*x*‐axis) and target (*y*‐axis). High value indicated that the expression (or proportion) of target could be significantly explained by the adjacent expression (or proportion) of the source. RMSE, root mean squared error of each model.

We linked single‐cell signals to the spatial transcriptome. Figures 3A,B and S4 show that OEE is present on the outer surface of the permanent tooth germ. *ALCAM*+ SC are found deeper, inside the OEE layer. In the permanent tooth germ, ameloblasts are few in number and located deep, near the stem cell layers. But in the primary tooth germ, they are abundant and closely packed. Ameloblasts are located at the boundary of pulp and epithelial tissues. The SI progenitors are located in the outer layer of ameloblasts. These patterns match the cell markers' spatial expression levels (Figure S5). We analysed the cell proportions in each spatial spot of all tissues (Figure 3C). We found IEE‐OEE mainly in permanent tooth germ and stratum intermedium mostly in 24 PCW tooth germ. Specifically, in 20‐week sample, we found that enamel knot cells were mainly distributed in the cusp region (Figure S4). This finding suggests that they might be secondary enamel knots.

Misty^22^ elucidated the spatial adjacency of different epithelial cell subtypes and key TFs. Figure 3D shows that when analysing epithelial cells, OEE and *ALCAM*+ SC had a close distribution with each other, while ameloblasts, enamel knots and stratum intermedium aggregated together. When including all cell types together, only mesenchyme showed close relationship with multiple epithelial cells. When analysing key TFs involved in epithelial differentiation (i.e., those TFs highlighted in Figure 2), we also observed that the expression levels of various key epithelial TFs was significantly associated to adjacent mesenchyme proportion (Figure 3D). In Conclusion, we inferred that the signal from mesenchyme cells had an important role in regulating dental epithelial differentiation. This process involved *DLX3*, which is known to be implicated in bone, hair and tooth development. Another example is *RUNX2*, a key factor in osteoblast differentiation and skeletal morphogenesis, which was found to be notably modulated by mesenchymal signals. In fact, the intercellular communication between mesenchymal and epithelial cells appears to employ a complex signalling cascade, ensuring the temporal and spatial control of these molecules during the various stages of tooth formation.

### 3.4 Involvement of *LHX9*, *TCF7* and *SP7* in dental pulp development

**FIGURE 4:**
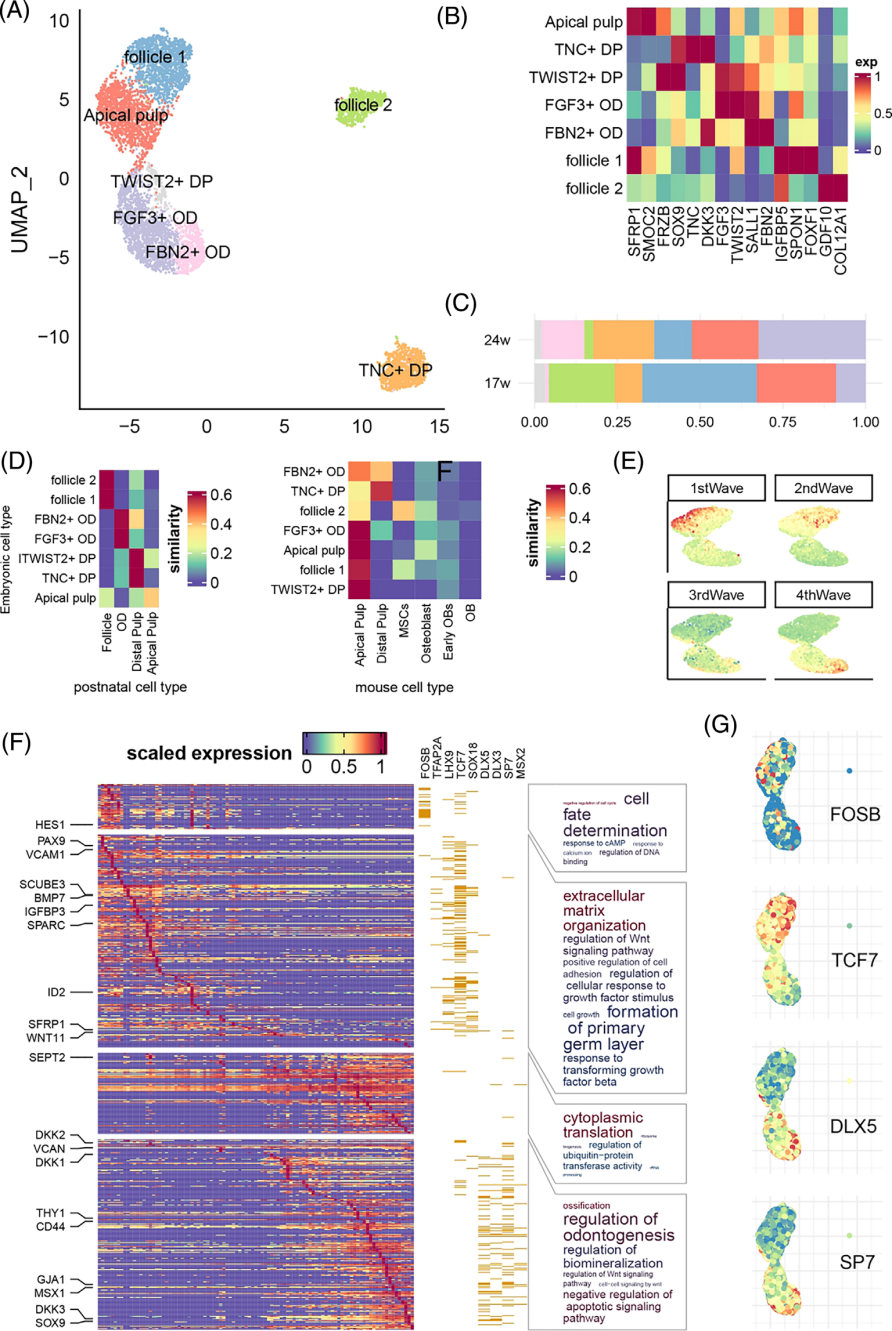
In‐depth analysis of dental mesenchyme. (A–C) similar to Figure 2A–C, but for epithelium clusters. Pseudotime trajectory was shown in Figure S7. AP, apical progenitor; MSC, mesenchymal stem cell; OD, pre‐odontoblast. (D) Comparison with previous dental cell atlas. Similarity was measured by Seurat integration score. (E) Expression of four waves of gene along mesenchyme development. (F) Heatmap of module gene expression along pseudotime. Yellow bar denoted that the gene was predicted to be target of the corresponding transcription factor. Word cloud showed GO term enriched by each wave. Font size denoted fold enrichment (large font size denoted larger fold enrichment), colour denoted enrichment *p*‐value. (G) Expression of selected regulons.

Similar to the analysis of dental epithelium, we further deciphered the spatiotemporal dynamics of mesenchyme cell groups. We identified seven subtypes of mesenchyme origin (Figures 4A–C and S6). Apical pulps (AP) were defined by the expression of *SFRP1*, *SOSTDC1* and *SMOC2*. We defined the distal pulp according to the expression of *SOX9*, which contained two subtypes: one group expressed *TNC*, *DKK3* and *HEY1* (*TNC*+ DP) and the other expressed *FRZB*, *FGF3* and *TWIST2* (*TWIST2*+ DP). We further defined two odontoblast groups by *SALL1* expression, and distinguished them by *FGF3‐TWIST2* expression (*FGF3*+ OD) and *DKK3‐FBN2* expression (*FBN2*+ OD). Lastly, we found two clusters of follicle cell, one expressed *IGFBP5* + *SPON1* + *FOXF1* (follicle 1) and the other expressed *GDF10* and *COL12A1* (follicle 2). Compared with 24‐PCW tooth germ, the 17‐PCW tooth germ had significantly larger proportions of follicle cells, and significantly smaller proportion of distal pulps (Figure 4C).

By integrating our data with the previous dental cell atlas from Hermans et al.^13^ (Figure 4D), we found that human embryonic dental mesenchyme was generally similar to postnatal cells: the follicle, odontoblast, and distal pulp subgroups were all closest to the same cluster in postnatal data. The only exception was apical pulp, which was moderately distinct from the postnatal apical pulp. Nonetheless, when comparing human dental cells with mouse dental cells, we found substantial differences between the two species. In fact, only *TNC*+ DP resembled the corresponding DP cluster in mouse data, while all other cell types were close to mouse apical pulp.

We further applied pseudotime analysis to reconstruct the developmental trajectory of the continuous cell clusters from follicle 1 to *FBN2*+ OD (Figure S7), and identified four waves of gene expression patterns along this trajectory (Table S8). As shown in Figure 4E, the first and second waves corresponded to progenitor cell differentiation, and the third and fourth waves corresponded to pulp cell development. The second wave peaked at the apical pulp population, where the pulp and follicle populations merged, resembling the segregation of the mouse dental follicle and dental papilla lineages discovered by Jing et al.^6^ The first wave included *HES1* and TWIST1, and was enriched in cell fate determination (GO *p*‐adjust = 2.28 × 10^−3^) and other processes related to cell cycle (Figure 4F). SCENIC^19^ analysis revealed that TFs regulating the first wave genes were immediate genes like FOS and JUN family like *FOSB* (Fisher test *p* = 3.60 × 10^−14^; Figure 4E and Table S9). The second wave of genes including *PAX9*, *SCUBE3* and *SPARC*, was found to be enriched in extracellular matrix organization (GO *p*‐adjust = 1.23 × 10^−18^) and formation of primary germ layer (GO *p*‐adjust = 8.38 × 10^−9^). They were also enriched in the predicted targets of *LHX9*, *TCF7* and *SOX18* (Fisher test *p* < 10^−10^; Figure 4F and Table S9). The third wave consisted of genes involved in cytoplasmic translation (GO *p*‐adjust = 7.66 × 10^−29^), without significant enrichment in specific TF regulon. Finally, the fourth wave genes, including *DKK2*, *VCAN* and *THY1*, were involved in the regulation of odontogenesis (GO *p*‐adjust = 1.68 × 10^−4^), Wnt signalling pathway (GO *p*‐adjust = 0.0001) and ossification (GO *p*‐adjust = 3.18 × 10^−6^). These genes are mainly regulated by TFs such as *DLX5*, *DLX3*, *SP7* and *MSX2* (Fisher test *p* < 1.78 × 10^−5^; Figure 4F and Table S9). The overall expression levels of these highlighted TFs were also in line with the four wave patterns (Figure 4G).

### 3.5 Pulp cell subtypes exhibited a distinct spatial distribution

**FIGURE 5:**
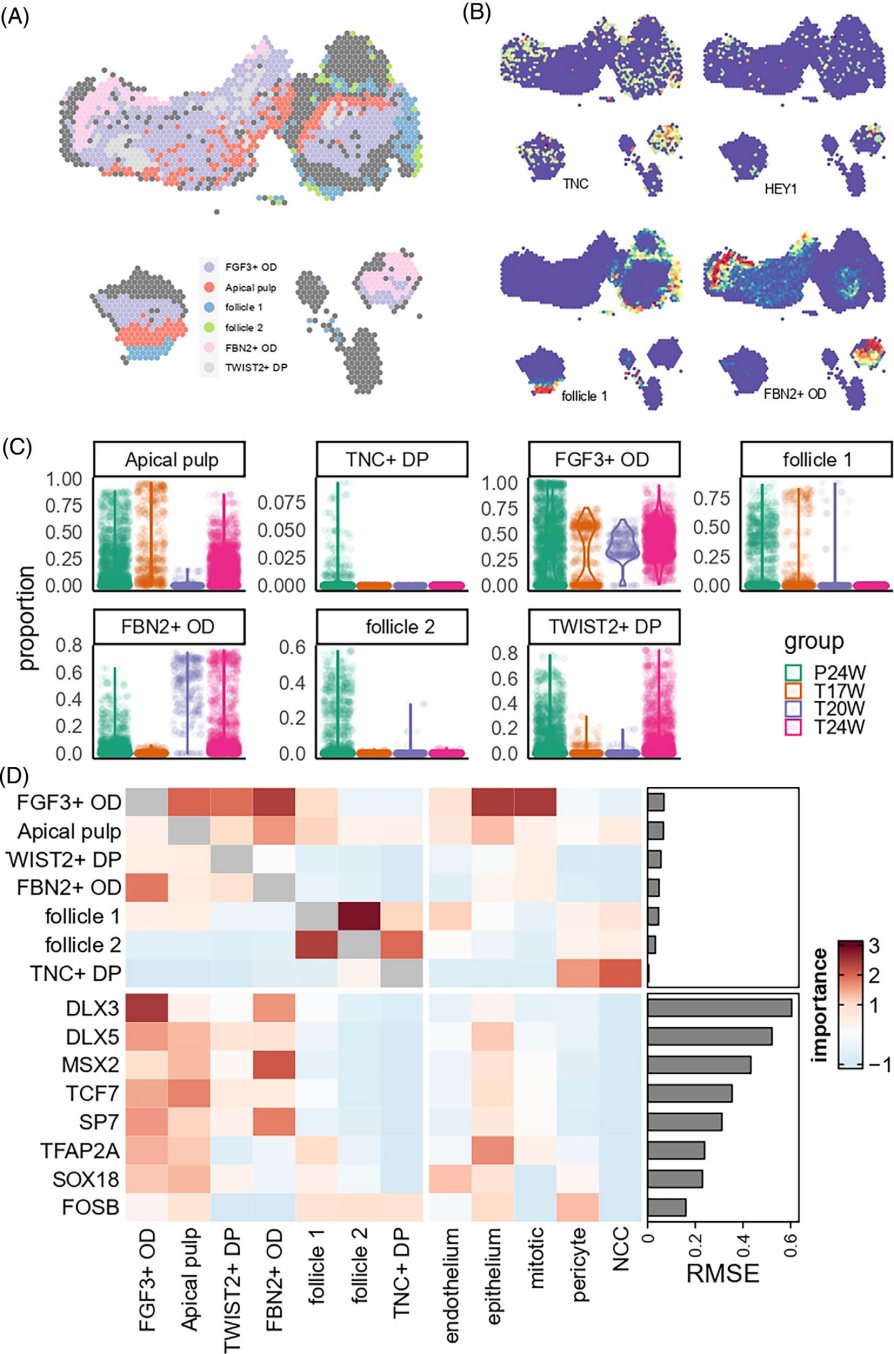
Spatial transcriptome of dental mesenchyme. (A) Cell type annotation of each spot of spatial transcriptomic. Spots not annotated as mesenchyme cell types were marked by dark grey. (B) Cell type probability spatial distribution and marker gene spatial expression. (C) Cell type probability distribution per developmental stage. P, primary teeth; T, permanent teeth. (D) misty result of spatial adjacency, quantified by model importance score. Higher importance score indicated that the two values were closer to each other on the spatial transcriptomic. RMSE, root mean squared error of each model.

We applied the Seurat anchor‐based integration algorithm^16^ to map single‐cell data to the spatial transcriptome (Figure 5A). In the 17 PCW primary tooth germ, we observed that follicle, distal pulp, apical pulp, and epithelium layers distributed from distal to proximal end, in line with their order in the developmental trajectory (Figure 4A). In the 24 PCW primary tooth germ, we further discovered that *FBN2*+ OD was at the outer layer of pulp cells, which consisted of *TWIST2*+ DP and *FGF3*+ OD. One exception was the sparsely distributed *TNC*+ DP in permanent tooth germ without significant spatial structure (Figures 5A and S8). We plotted the expression of its marker *TNC* and *HEY1* and also found no matching patterns (Figure 5B). We inferred that *TNC*+ DP was loosely distributed in all regions of pulp tissues. As a comparison, the density as well as the marker expression of other mesenchymal cell subtypes (Figures 5B, S8 and S9) were all aggregated to specific regions. The temporal distribution (Figure 5C) showed that *TNC*+ DP and two follicle and subgroups were mainly found in immature permanent tooth germ (*p* < 10^−10^), whereas odontoblast and pulp cells were mainly found in mature primary tooth germ (*p* < 10^−10^).

We also applied misty^22^ to quantify the spatial adjacency among cell subtypes and key TFs (Figure 5D). Within mesenchyme cell groups, we found a close relationship between two subgroups of follicle cells (misty adjacency score >2.5) as well as *FGF3*+ OD and *FBN2*+ OD (misty adjacency score >2). Apart from mesenchyme cell groups, only epithelium and mitotic cells had a close relationship with pulp cells (adjacency score >2). For TFs, pulp cells had close association with nearby expression values of multiple TFs that played important roles in mesenchyme differentiation, except for *FOSB* which took effect in early progenitors. Another spatial association was found between *FBN2*+ OD and *MSX2* gene expression (adjacency score = 2.02).

### 3.6 Signalling network analysis of dental development

**FIGURE 6:**
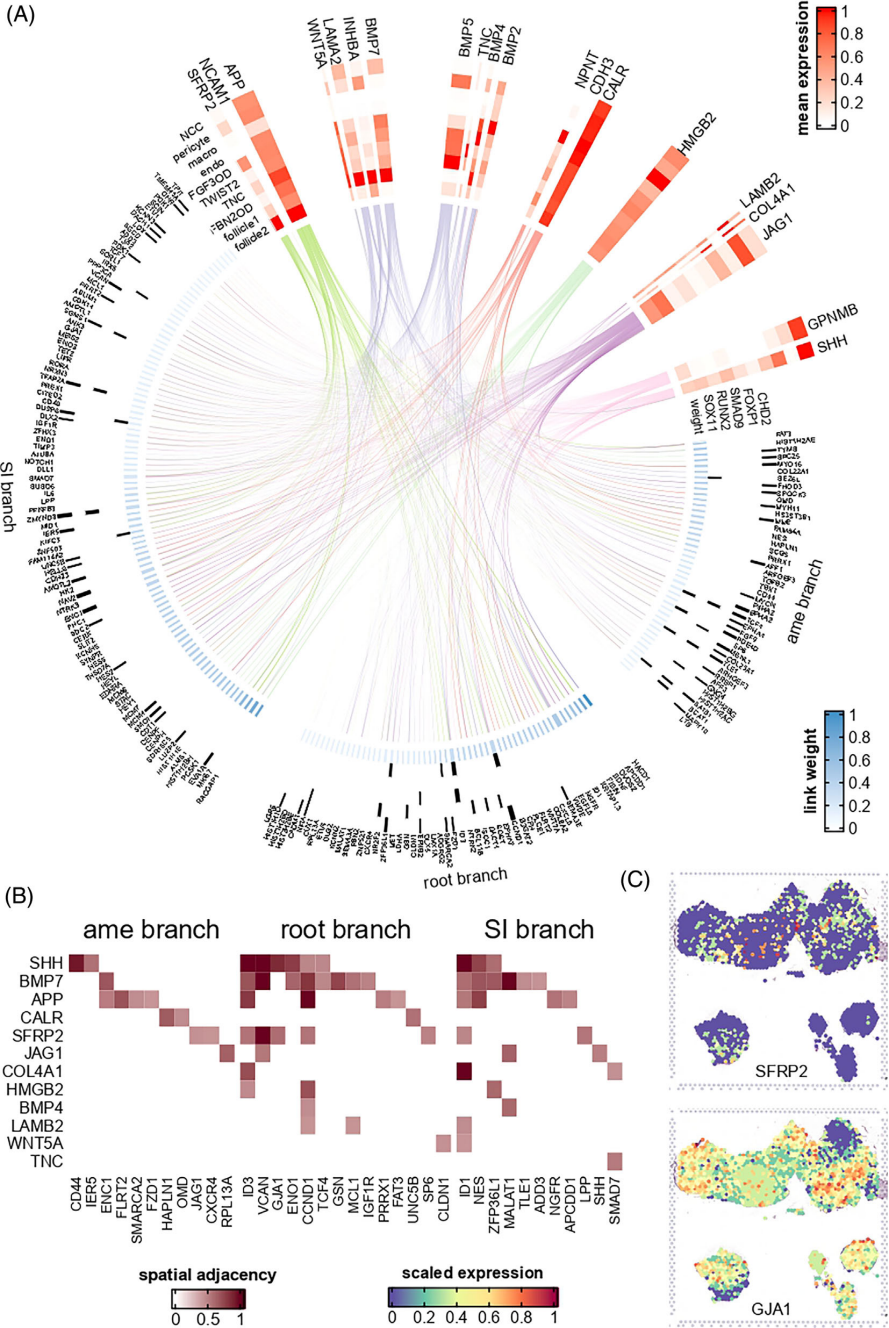
Signalling network regulating epithelium development. (A) Each line linked a ligand and a predicted target; line colour showed the cell type in which the ligand had the highest expression (corresponded to colour bar of Figure 1B). Heatmap showed cell type average expression of ligands. Black bar denoted the transcription factor that was predicted to regulate the corresponding target gene. Link weight was calculated by nichenetr. (B) Spatial adjacency between ligand and target, quantified by misty model importance score. (C) Spatial expression of selected ligand and target.

After annotating the refined cell subtypes of the embryonic tooth germ, we were able to study the intercellular signalling pathways that regulate dental development. We first applied nichenetr^21^ on scRNA data and misty^22^ on ST data to build a signalling network that regulates a branch‐specific key genes in epithelial development (181 key genes included in nichenetr; Figure 6A). By integrating the prioritized upstream ligands of the both root branch, SI branch and ameloblast branch, it was found that signals from the mesenchyme (including follicle cells) and pericytes regulated the largest number of epithelial key genes (*n* = 142 and 92, respectively, Figure 6 and Table S10). The top credible ligands, including *BMP5*, *SFRP2*, *JAG1* and *HMGB2* (Figure 6B,C and Table S11), were identified. Among them, *JAG1* was predicted to regulate the largest number (*n* = 91) of key genes in epithelial differentiation, including *GJA1*, *NOTCH1*, and so on. Two notable exceptions were *GPNMB* and *SHH*: they were the only prioritized ligands that showed the highest expression in ameloblasts (Figure S10) and had high potential to regulate SI and ameloblast branch development (AUC = 0.59 and 0.53, respectively), suggesting that they might act through autocrine or paracrine signalling. Despite their expression in epithelium, they were also found to be expressed in NCC (Figure S10).

**FIGURE 7:**
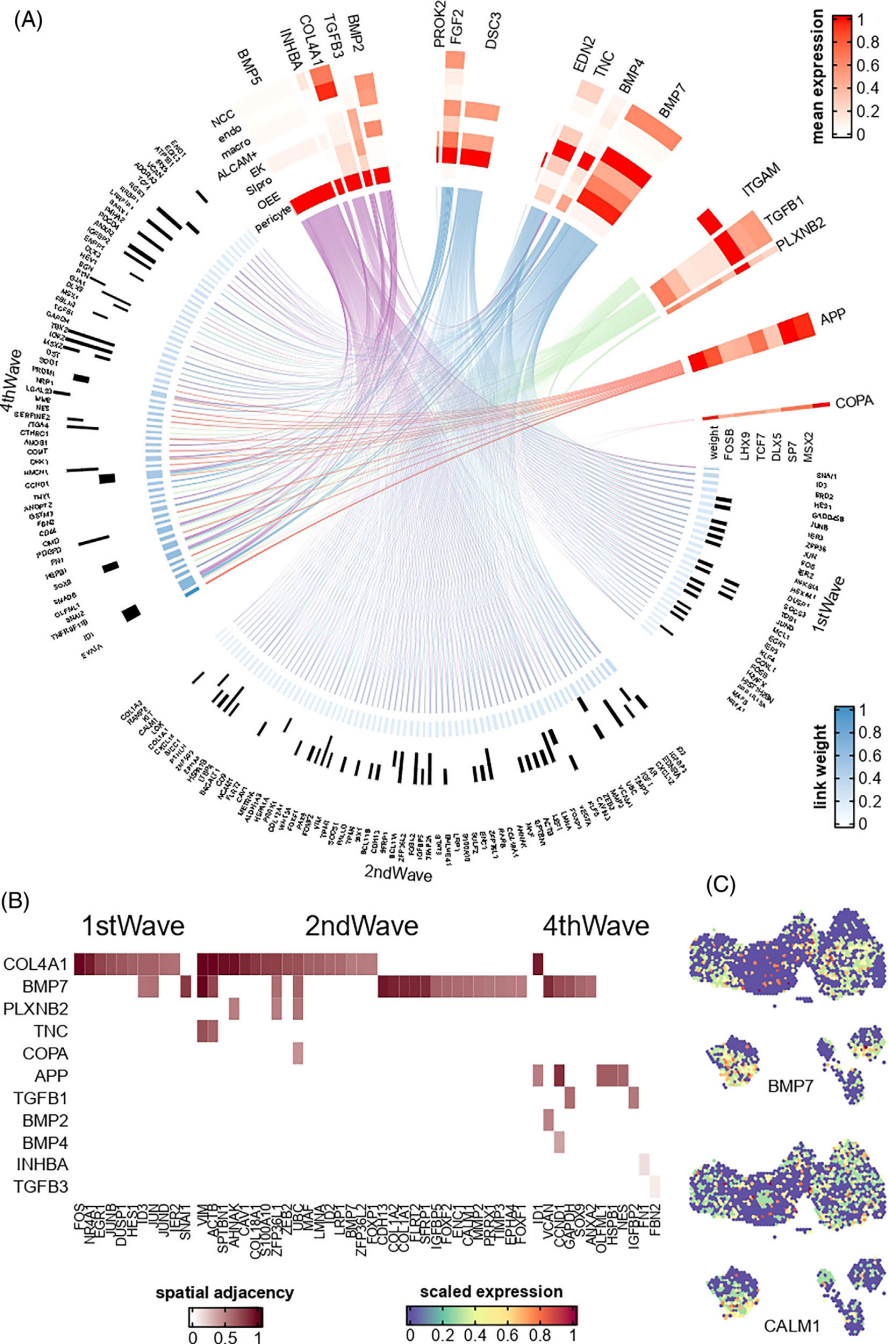
Signalling network regulating mesenchyme development. (A) Each line linked a ligand and a predicted target; line colour showed the cell type in which the ligand had the highest expression (corresponded to colour bar of Figure 1B). Heatmap showed cell type average expression of ligands. Black bar denoted that the transcription factor of this row was predicted to regulate the corresponding target gene. Link weight was calculated by nichenetr. (B) Spatial adjacency between ligand and target, quantified by misty model importance score. (C) Spatial expression of selected ligand and target.

Finally, we analysed the intercellular signalling networks that regulate dental mesenchyme development. For all 162 mesenchyme key genes (genes of the first, second and fourth waves of Figure 4D), 135 were regulated by ligands from pericytes, and 133 were regulated by ligands from epithelium (Figure 7). These included ligands such as *BMP5*, *BMP7*, *DSC3*, *COL4A1* and so on. Since various lines of evidences predicted that *COL4A1* was interacted with multiple mesenchymal trajectory genes, considering its known role in the basement membrane and angiogenesis, we hypothesized that *COL4A1* might regulate the spatial distribution of mesenchyme cells. By applying misty, we found that *COL4A1* was indeed spatially adjacent to the apical pulp (*p* < 2.2 × 10^−16^), supporting our hypothesis. We proposed that the high expression of *COL4A1* represented the formation of the endothelial and vascular niche, which is vital for mesenchyme differentiation.^24^ We also found that different ligands tended to regulate different stages of mesenchyme development. For example, *BMP5* and *BMP7* regulated all first‐wave genes and over 70% of the second‐wave genes that were included in the nichenetr analysis, whereas all predicted targets of *APP* (mainly expressed in endothelium) and *TGFB1* (mainly expressed in macrophage) belonged to the fourth wave (Figure S11 and Table S12). In general, upstream signalling pathways of mesenchyme development were restricted to a few vital ligands and cell types, such as BMP signals from pericyte and epithelium. Finally, we showed that the spatial distribution of these signalling molecules was in accordance with their predicted functions, as detailed in Supplementary Materials in Data S1; Table S13; Figures S12 and S13.

## 4 DISCUSSION

In this current study, we applied single‐cell RNA sequencing and spatial transcriptome analysis to illustrate the spatiotemporal cell landscape of human tooth development. We revealed the cellular composition and spatial distribution of human embryonic tooth germ across various developmental periods and tissues from both primary and permanent tooth germ. We also depicted the developmental trajectory, biological insights, essential transcription factors and signalling pathways of dental epithelium and mesenchyme development. In complement with the existing dental cell atlases of prenatal and postnatal mouse tooth,^6^, ^7^ postnatal human teeth^7^, ^9^ and teeth germ,^12^ our result brought one more step closer to a complete single‐cell picture of dental development.^13^

Our scRNA and ST data of human embryonic dental tissues have further promoted the cell atlas of the early developmental process of the human tooth. The majority of currently available single‐cell data on human teeth^13^ comes from young volunteers, that have missed progenitor cells at an early stage. Only a few recent scRNA studies have focused on human foetal dental tissue^15^ providing remarkable novel insights into tooth development. However, there are still gaps in our understanding of specific cell types and developmental stages, such as mesenchyme cells and early PCW. On the other hand, studies based on mouse embryonic tissues may not be directly applicable to human research due to significant cross‐species differences. Rodent incisors have a relatively short lifespan and are routinely lost. Many cell types, including ameloblasts and odontoblasts that secrete mineralized enamel and dentin, respectively, originate from self‐renewal cells located in the proximal part of the tooth.^25^ Some of the vital structures of this process, such as the transit‐amplifying (T‐A) zone, were not found in human dental tissue.^26^, ^27^, ^28^ Thus, it is desirable to generate the dental cell landscape for both human and mice at the developing stage to achieve an intact understanding of human dental generation.

Our result suggested that many potential signalling molecules existed between epithelial cells and mesenchyme cells, which might mediate their crosstalk and regulated the developmental process of each other via intercellular signalling networks.^29^ In the mouse incisor, *BMP‐SMAD‐SHH* signals from a transient structures Hertwig's epithelial root sheath^30^ interacts with mesenchymal cells and regulate root elongation and epithelium maturation.^31^ In an autocrine manner, SHH is produced by the dental epithelial cells themselves and acts on the same cells, influencing their proliferation, differentiation, and survival during tooth development. This self‐stimulation ensures proper growth and patterning of the dental epithelial tissue.^32^ Additionally, SHH functions in a paracrine manner, where it is secreted by the dental epithelial cells and diffuses to nearby mesenchymal cells. The interaction between SHH and mesenchymal cells induces signalling cascades that direct the formation and differentiation of various dental structures, such as the dental papilla and enamel organ.^33^ The epithelium could also regulate the mineralization of nearby dentin although the secretion of signal molecules,^34^ including Wnt and fibroblast growth factors.^35^ In line with these observations, previous scRNA intercellular signalling network analyses on postnatal pulp^10^ and tooth germ^12^ tissues have demonstrated the existence of dense communication. Our work extends this knowledge to the prenatal epithelium–mesenchyme developmental process and highlights more signal pathways such as *JAG1*, *SFRP2* and *COL4A1*.

In this study, we focus on a narrow developmental window (17–24 PCW). Enamel organs mature after 12 PCW, and then the odontoblasts start forming the dentin. We selected foetal tooth germs from 17 to 24 PCW because tooth germs of younger gestational age are not yet fully developed and are relatively small, making it difficult to obtain samples. On the other hand, tooth germs of older gestational age have already formed a considerable amount of dental hard tissue, and it is necessary to remove this hard tissue during sampling, resulting in the loss of a portion of the tooth germ tissue.

Our study has limitations. Early embryos (<12 PCW) have not yet formed a fully mature tooth germ tissue, while later embryonic healthy tissues (>28 PCW) are difficult to access due to ethnical concerns. The timepoints between 17 and 24 PCW are also not fully covered, which might show specific differences compared with our samples. Thus, dental developmental processes at these stages could not be covered in the current study. It is desirable to build more comprehensive cell atlas covering more timepoints in the future. Our relatively small sample size could not provide a comprehensive coverage of the entire developmental trajectory. The spatial structures in the third dimension are incomplete across various slicing procedures, leaving gaps that beckon for future endeavours to be devoted to their meticulous completion.

In conclusion, we have constructed a spatiotemporal cell landscape of human embryonic tooth germ, depicted the developmental trajectory of the dental epithelium and mesenchyme, and revealed the biological mechanisms underlying these processes. Our results served as a foundation for future research on dental development and stem cell engineering.

## AUTHOR CONTRIBUTIONS

Yueqi Shi designed the study. Wenjia Wei and Jin Qiu supervised the study. Jin Qiu and Li Han collected and pre‐processed the sample. Yueqi Shi, Yejia Yu, Shaoyi Wang, Ke Guo, Jingang Yang, Jutang Li and Shoufu Sun performed the experiments. Yueqi Shi analysed the data and drafted the manuscript. All authors read, revised and approved the manuscript.

## FUNDING INFORMATION

This work was supported by Research Fund of Shanghai Tongren Hospital, Shanghai Jiaotong University School of Medicine (No: TRYJ2021JC14, TRKYRC‐xx202207).

## CONFLICT OF INTEREST STATEMENT

The authors declared no competing interest.

## Supporting information


**Table S1.** Markers of major cell types calculated by FindVariableGenes function.

**Table S2.** Gene modules of epithelial root branch calculated by monocle3.

**Table S3.** Gene modules of SI branch calculated by monocle3.

**Table S4.** Gene modules of ameloblast branch calculated by monocle3.

**Table S5.** Enrichment of root branch gene module in transcription factor targets predicted by SCENIC.

**Table S6.** Enrichment of SI branch gene module in transcription factor targets predicted by SCENIC.

**Table S7.** Enrichment of ameloblast branch gene module in transcription factor targets predicted by SCENIC.

**Table S8.** Gene modules of mesenchyme development calculated by monocle3.

**Table S9.** Enrichment of mesenchyme development gene module in transcription factor targets predicted by SCENIC.

**Table S10.** Ligand‐target predictions of epithelial branch genes by nichenetr.

**Table S11.** Ligand importance predictions of epithelial branch genes by nichenetr.

**Table S12.** Ligand‐target predictions of mesenchyme development genes by nichenetr.

**Table S13.** Ligand importance predictions of mesenchyme development genes by nichenetr.

**Data S1.** Supporting Information.

## Data Availability

Data could be downloaded at 10.17632/v3wgx8pm5y.1.
